# Up-regulation of long non-coding RNA XLOC_010235 regulates epithelial-to-mesenchymal transition to promote metastasis by associating with Snail1 in gastric cancer

**DOI:** 10.1038/s41598-017-02254-6

**Published:** 2017-05-26

**Authors:** Yu-yi Liu, Ze-hong Chen, Jian-jun Peng, Jia-lin Wu, Yu-jie Yuan, Er-tao Zhai, Shi-rong Cai, Yu-long He, Wu Song

**Affiliations:** Department of Gastrointestinal Surgery, The First Affiliated Hospital, Sun Yat-sen University, Zhongshan Second Road 58, Guangzhou, 510080 Guangdong Province China

## Abstract

We previously performed long non-coding RNA (lncRNA) expression microarray analyses to identify novel indicators for gastric cancer (GC) metastasis and prognosis in which we identified lncRNA XLOC_010235 (XLOC) as a candidate RNA. However, XLOC_010235 molecular mechanism of action remains unclear. Gain and loss of function approaches were used to investigate the biological role of XLOC *in vitro*. The effects of XLOC on cell viability were assessed by CCK-8 proliferation assays. Real-time PCR, western-blot and immunofluorescence were used to evaluate the mRNA and protein expression of Snail and multiple EMT related molecules. The positive XLOC/Snail1 interaction was identified and verified by immunohistochemistry assay and bivariate correlation analysis. Ectopic expression of XLOC facilitate cell viability, migration and invasion, leading to the acceleration of metastasis, while depletion of XLOC expression hindered cell migration and invasion. Moreover, over-expression of XLOC was found to play a important role in epithelial-to-mesenchymal transition (EMT) through the regulation of E-cadherin, N-cadherin and Vimentin expression, in which transcriptional factor Snail1 was involved. These results advance our understanding of the role of lncRNA XLOC_010235 as a active regulator of EMT by associating with Snail1, which may help in the development of new therapeutics.

## Introduction

Gastric cancer (GC) is one of the most prevalent malignant tumors, with a global incidence that ranks fourth among all tumor types. Although the global mortality from gastric cancer has decreased over the past several decades, mortality is extremely high in the West, with many patients diagnosed with an advanced TNM stage tumor and a poor prognosis^1^. However, traditional prognostic assessment based on TNM stage and histological differentiation is not yet able to indicate the metastatic status of a tumor or to predict an individual’s clinical outcome. As a result, patients with the same stage tumor may exhibit different outcomes. Thus, there is a need to explore novel prognostic biomarkers to assess the metastatic process.

Over the past decade, the key role of epithelial-mesenchymal transition (EMT), a biological process where epithelial cells lose their polarity and transit into a mesenchymal phenotype, have been elucidated in cancer cell metastasis^2^. This process is characterized by the loss of epithelial markers and a gain in mesenchymal markers^3, 4^. E-cadherin expression and N-cadherin or Vimentin expression are considered to be the most important molecular markers of EMT^5^. Several EMT inducers, including transcription factors such as Snail (Snai1), Slug (Snai2), ZEB1 and ZEB2/Sip1have been shown to function by inhibiting E-cadherin expression^6^. Snail is considered to be the most potent transcriptional suppressor of E-cadherin, since for example mice deficient in Snail expression fail to down regulate E-cadherin^6^. The suppression of Snail by siRNA-treatment or anti-senseoligos has been shown to increase E-cadherin expression andinhibit tumorigenesis and EMT in different animal models^7, 8^.

Until now, substantial effort has been devoted to reveal the modulation of EMT in cancer metastasis. It has been proved that EMT can be initiated by external signals, such as hepatocyte growth factor (HGF), epidermal growth factor (EGF), transforming growth factor (TGF)-b, and fibroblast growth factor (FGF)^9^. Besides these signaling pathways activated by membrane receptors, importance have been attached to non-coding RNAs in the regulation of the epithelial phenotype by controlling EMT inducers. The miR-200 family has been found to control EMT by down regulating the expression of Zeb factors^10^. Furthermore, the long non coding RNA (lncRNA) MALAT-1 promoted EMT by regulating ZEB1, ZEB2 and Slug expression, and activating Wnt signaling^11^.

More recently, several reports have shown that long non-coding RNAs (lncRNAs), which have emerged as a key players with greater than 200 nucleotides in length in three types non-coding RNAs, have been implicated in tumorigenesis, cancer progression, and metastasis^12–14^. LncRNAs are usually expressed in a spatially and temporally specific manner during cell development shown a cell-, tissue- and development specific model^15^. LncRNAs may function as oncogenes or tumor suppressors by altering chromatin structure or by regulating the transcription of protein-coding genes^16–18^. Therefore, identification of cancer-associated lncRNAs and investigation of their molecular and biological functions in controlling EMT are important in understanding the molecular biology of GC metastasis and progression.

XLOC_010235, an 302-bp lncRNA on chromosome 12 was firstly identified by Cabili *et al*. XLOC_010235 is verified to over-expressed in gastric cancer cells and closely related to tumor metastasis and patient prognosis in our previous study^19^. In current study, we investigated the effects of XLOC expression on GC cell phenotypes *in vitro* and demonstrated that ectopic expression of XLOC can significantly influence expression levels of multiple EMT related molecules including E-cadherin, N-cadherinand and Vimentin, which indicated that XLOC affected invasion and metastasis partly through epithelial-mesenchymal transition. Moreover, our findings showed that transciptional factor Snail1 were involved in the XLOC-mediated promotion of cell migration and invasion via EMT. All these results pay the way for further investigation about signal pathway implicated in the interaction of XLOC with Snail1 and suggest XLOC-010235 may serve as an indicative marker for the GC metastasis.

## Results

### XLOC promotes gastric cancer cell proliferation and exhibits an significant effect on gastric cancer cell migration and invasion *in vitro*

**Figure 1:**
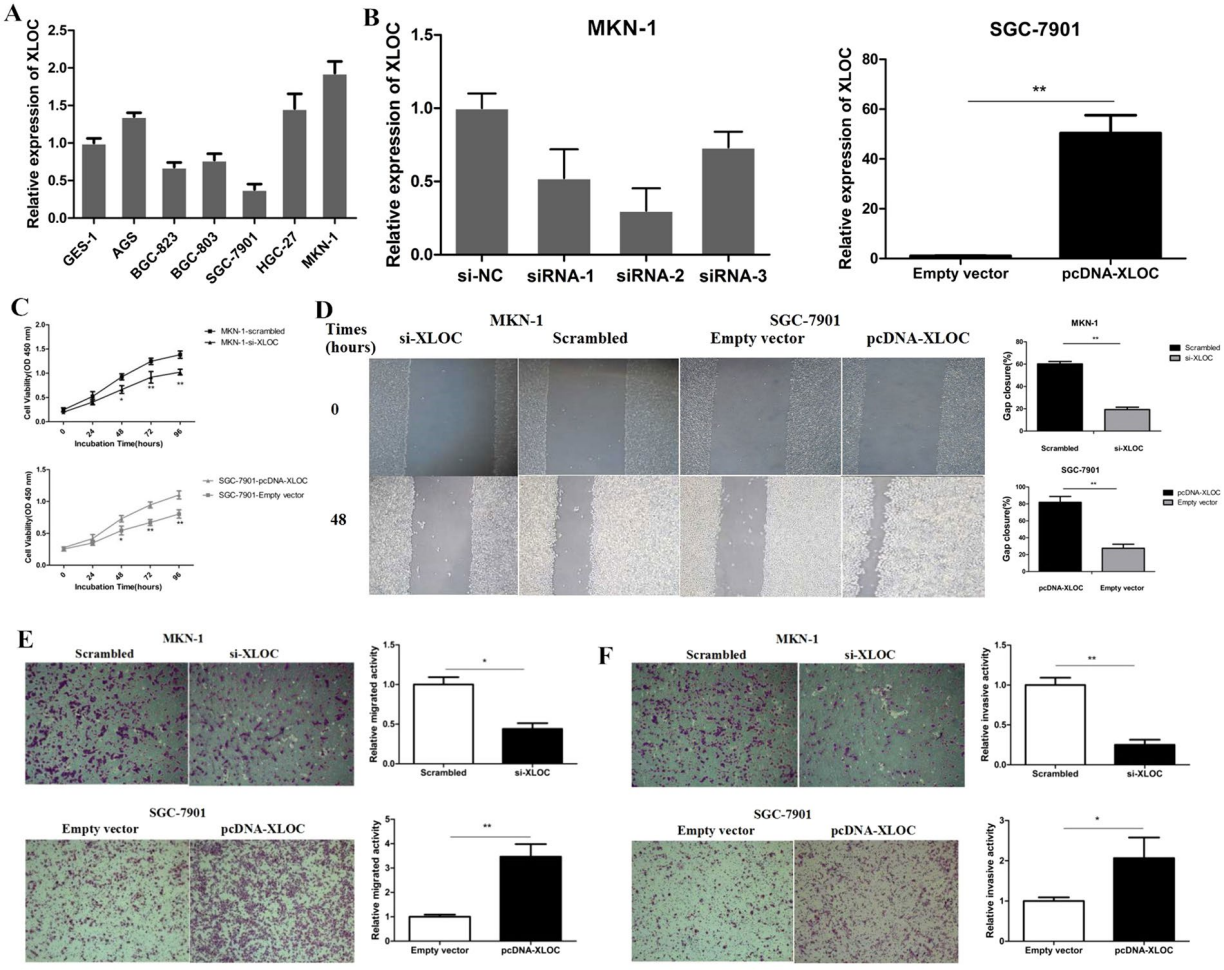
Effects of XLOC_010235 on gastric cancer cell migration and invasion *in vitro*. SGC-7901 cells were transfected with pcDNA-XLOC_010235, and MKN-1 cells were transfected with si-XLOC_010235. (**A**) XLOC_010235 expression levels of GC cell lines (AGS, BGC-823, BGC-803, SGC-7901, HGC-27, MKN-1) compared with that in normal human gastric epithelial cells (GES-1). (**B**) q-PCR analysis of XLOC_010235 expression levels following the treatment of MKN-1 cells with scrambled siRNA and si-XLOC_010235 (left panel), and the treatment of SGC-7901 cells with empty vector and pcDNA-XLOC_010235 (right panel). (**C**) Forty-eight hours after transfection, CCK-8 proliferation assays were conducted to determine the proliferation of MKN-1 and SGC-7901 cells. (**D**) Wound healing assays were performed to investigate the migratory ability of gastric cancer cells. (**E**,**F**) Transwell assays were used to investigate the changes in the migratory and invasive abilities of gastric cancer cells. All experiments were performed in triplicate. Bars: SD. Bars: SD; *p < 0.05 and **p < 0.01.

As our previous study has validate the association of XLOC and GC metastasis on clinic, we further evaluated the biological role of XLOC in GC cell proliferation, migration and invasion. In a series of gastric cell lines, MKN-1 was selected for knockdown of XLOC because it exhibits a higher expression of XLOC. In addition, over-expression of XLOC was performed by transfecting pcDNA3.1-XLOC vector into SGC-7901 for its relatively low level of XLOC expression (Fig. 1A). The over-expression and knockdown of XLOC was verified by qRT-PCR (Fig. 1B). CCK-8 proliferation assay results showed that in both MKN-1 and SGC-7901 cell lines XLOC exhibits an significant effect on cell viability (Fig. 1C). Subsequently, we investigated the effect on cell migration and invasion. The wound healing assay results showed that SGC-7901 cells transfected with pcDNA-XLOC resulted in a faster closing of scratch wounds (migration promotion) compared with that for control cells. Moreover, MKN-1, which have a naturally high XLOC expression, after knockdown of XLOC, exhibited a lower scratch closure rate (migration inhibition) than control cells (Fig. 1D). We then observed cancer cell invasion through matrigel, and migration through transwells. As shown in Fig. 1E,F, reduced XLOC expression levels significantly hindered the migration and invasion of MKN-1 cells compared with controls (P < 0.05). Parallelly, mobility and invasiveness of SGC-7901 cells were also promoted following up-regulation of XLOC expression (P < 0.05; Fig. 1E,F). These findings demonstrate that XLOC may be closely related to the migration and invasion of GC cells.

### XLOC impacts GC cell EMT

**Figure 2:**
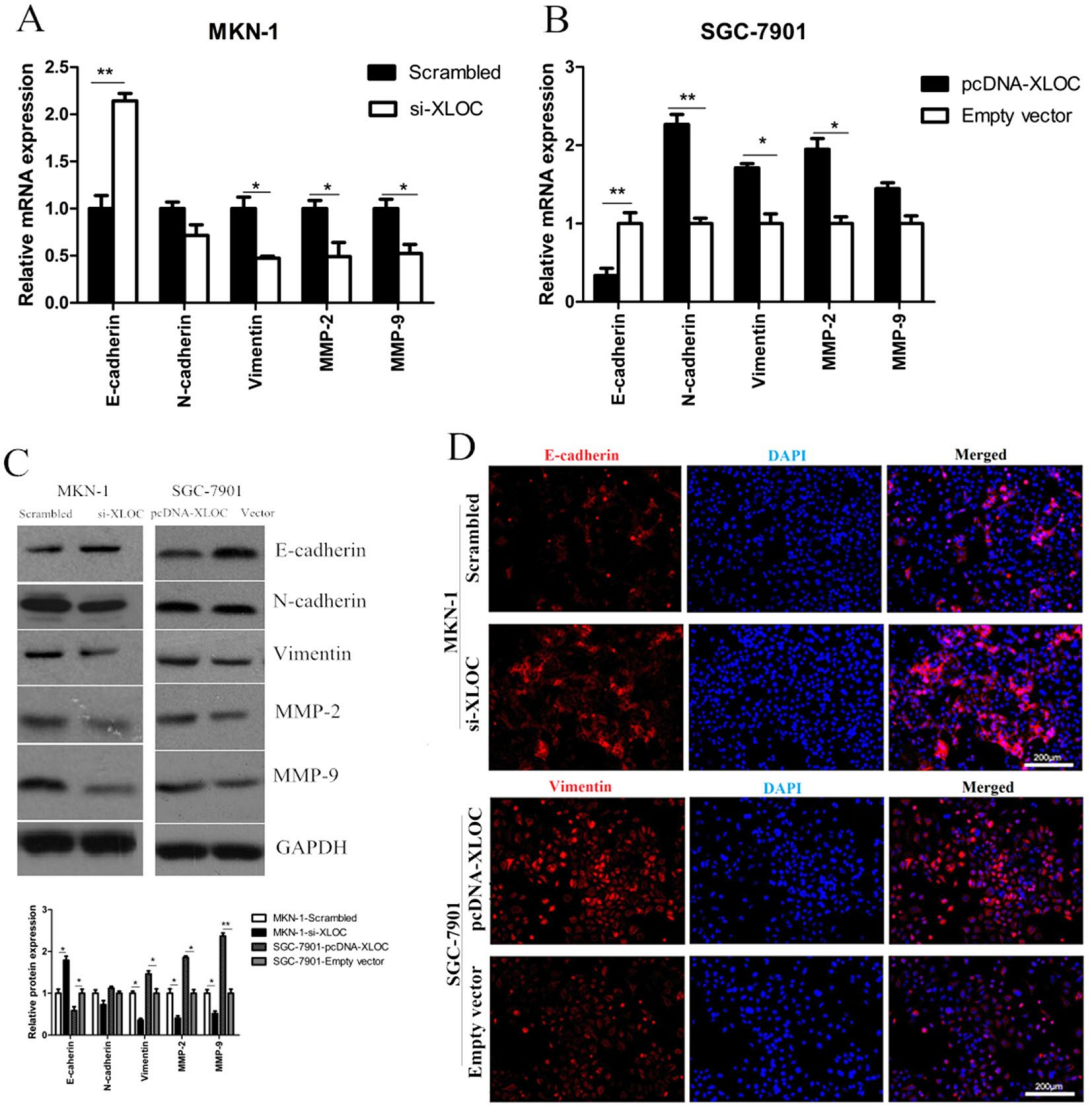
Over-expression of XLOC_010235 promotes GC cell invasion and metastasis by affecting EMT. (**A**,**B**) Analysis of E-cadherin, N-cadherin, Vimentin, MMP-2 and MMP-9 expression following the treatment of MKN-1 cells with scrambled si-RNA and si-XLOC_010235, and the treatment of SGC-7901 cells with empty vector and pcDNA-XLOC_010235 by q-PCR. **(C**,**D)** Analysis of E-cadherin and Vimentin expression in MKN-1 cells treated with si-XLOC and SGC-7901 cells treated with pcDNA-XLOC by western blot and immunofluorescence. All experiments were performed in triplicate. Bars: SD. Bars: SD; *p < 0.05 and **p < 0.01.

Q-PCR and western blotting assays were performed to detect the expression of EMT-induced markers (E-cadherin, N-cadherin and Vimentin) in cells with over-expression and knockdown of XLOC. our findings revealed that up-regulation of XLOC expression led to increased N-cadherin, Vimentin, MMP-2 and MMP-9 expression with a reduced expression of E-cadherin. Reversely, decreased XLOC expression levels induced E-cadherin expression while decreased N-cadherin, Vimentin, MMP-2 and MMP-9 expression (Fig. 2A,B). Moreover, western blotting and immunofluorescence analysis also showed that repressed XLOC expression stimulated E-cadherin expression and reduced Vimentin expression in GC cells, while enhanced XLOC expression exhibited contrary results (Fig. 2C,D).

### Up-regulated expression of Snail1 is implicated in the XLOC-mediated promotion of EMT

**Figure 3:**
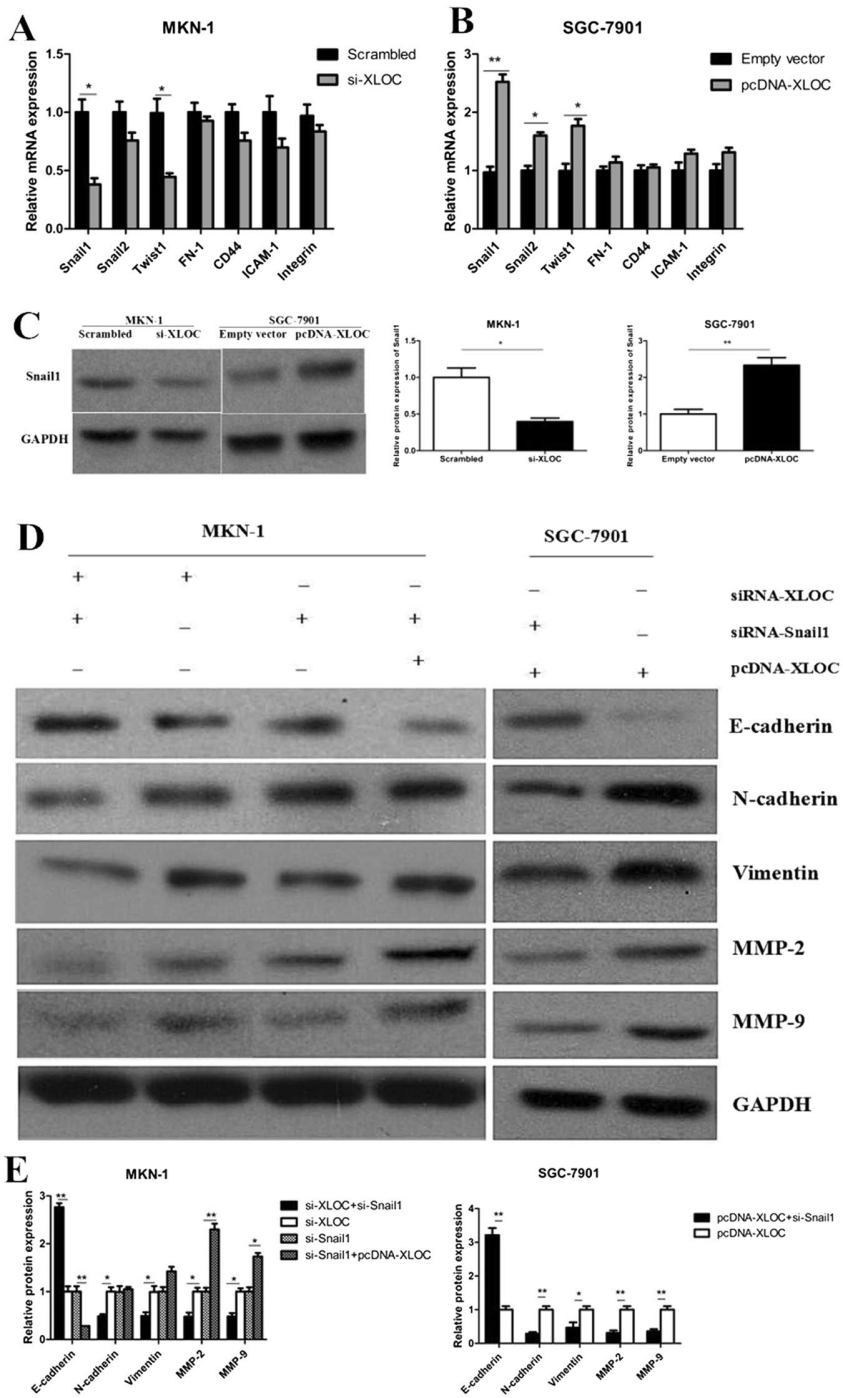
XLOC_010235 regulates Snail1 expression in affecting EMT. (**A**) Expression of EMT-related molecules (Snail1, Snail2, Twist1, FN-1, CD-44, ICAM-1, Integrin) detected using qRT-PCR after XLOC_010235 was blocked in MKN-1 cells or (**B**) over-expressed in SGC-7901 cells. (**C**) After XLOC_010235 expression is blocked in MKN-1 cells, western blot analysis shows that the Snail1 protein level is diminished, as compared to the level in the control group (left panel). The Snail1 protein level is elevated after XLOC_010235 is over-expressed in SGC-7901 cells, as compared to the level in the control group (right panel). (**D**,**E**) Analysis of E-cadherin, N-cadherin, Vimentin, MMP-2 and MMP-9 expression in MKN-1 cells following the treatment with si-XLOC_010235+ si-Snail1 compared to groups treated with si-XLOC_010235 alone, the treatment with pcDNA-XLOC_010235 + si-Snail1 compared to groups treated with si-Snail1 alone and in SGC-7901 cells following the treatment with pcDNA-XLOC_010235 alone compared to groups treated with pcDNA-XLOC_010235 + si-Snail1 by western blot. All the above experiments were performed in triplicate. Bars: SD; *p < 0.05 and **p < 0.01.

To further explore the molecular mechanisms by which XLOC contributes to the phenotypes of gastric cancer cells, we inspected potential targets involved in tumor invasion and metastasis. By using qRT-PCR, we originally detected the host gene FOXF1, which plays an essential role in cancer cell invasion and migration^20^, in both XLOC over-expressing cells and XLOC-knockdown cells compared with control cells. However, no changed expression was found.(data not shown) The results suggested that XLOC may not exert its effects by modulating its host gene. As cell adhesion molecules and numerous transcription factors are involved in invasion and metastasis in various cancers, we then performed qRT-PCR to monitor the expression of cell adhesion molecules and transcription factors that have been confirmed to be implicated in tumor migration and invasion (such as Snail1, Snail2, Twist1, fibronectin1[FN1], Integrin, CD44, ICAM-1). Among them, Snail1 was found to be significantly altered. When XLOC was over-expressed or blocked, mRNA of Snail1 was reduced by approximately 60% or increased 2.5-fold, respectively, compared to the control groups (Fig. 3A,B). Furthermore, it is universally acknowledged that Snail1 is closely associated with tumor migration and invasion for involvement in the EMT. Therefore, we performed western blot analysis to sequentially validate the expression of the Snail1 on protein level. The Snail1 protein expression was also diminished by 54% in MKN-1 cells transfected with si-XLOC and was elevated more than 2.0-fold after transfection with pcDNA-XLOC (Fig. 3C) compared to the respective control cells. Our findings show that Snail1 is positively regulated by XLOC at the mRNA and protein levels.

To uncover whether Snail1 was involved in the XLOC-induced promotion of EMT related to metastatic phenotype in gastric cancer cell, we further implemented western blot assays. The knockdown effect of si-Snail1 was confirmed by qRT-PCR (Supplementary file 3. Fig. S1). After transfection with si-XLOC, MKN-1 cells were co-transfected with si-Snail1. We found that co-transfection of si-Snail1 could availably alleviate the metastatic phenotype and suppress the EMT process (Fig. 3D,E). Simutaneously, we co-transfected MKN-1 cells with pcDNA-XLOC after transfection of si-Snail1. The co-transfection of pcDNA -XLOC absolutely exerts influence on the promotion of EMT compared to the alone transfection of si-Snail1. In addition, EMT indicators were detected in SGC-7901 cells with XLOC over-expression alone and XLOC plasmid + siRNA Snail1 (Fig. 3D,E). These data indicated that Snail1 was positively involved in the XLOC-induced promotion of EMT procedure.

### Positive correlationship between the expression of Snail and XLOC_010235

**Figure 4:**
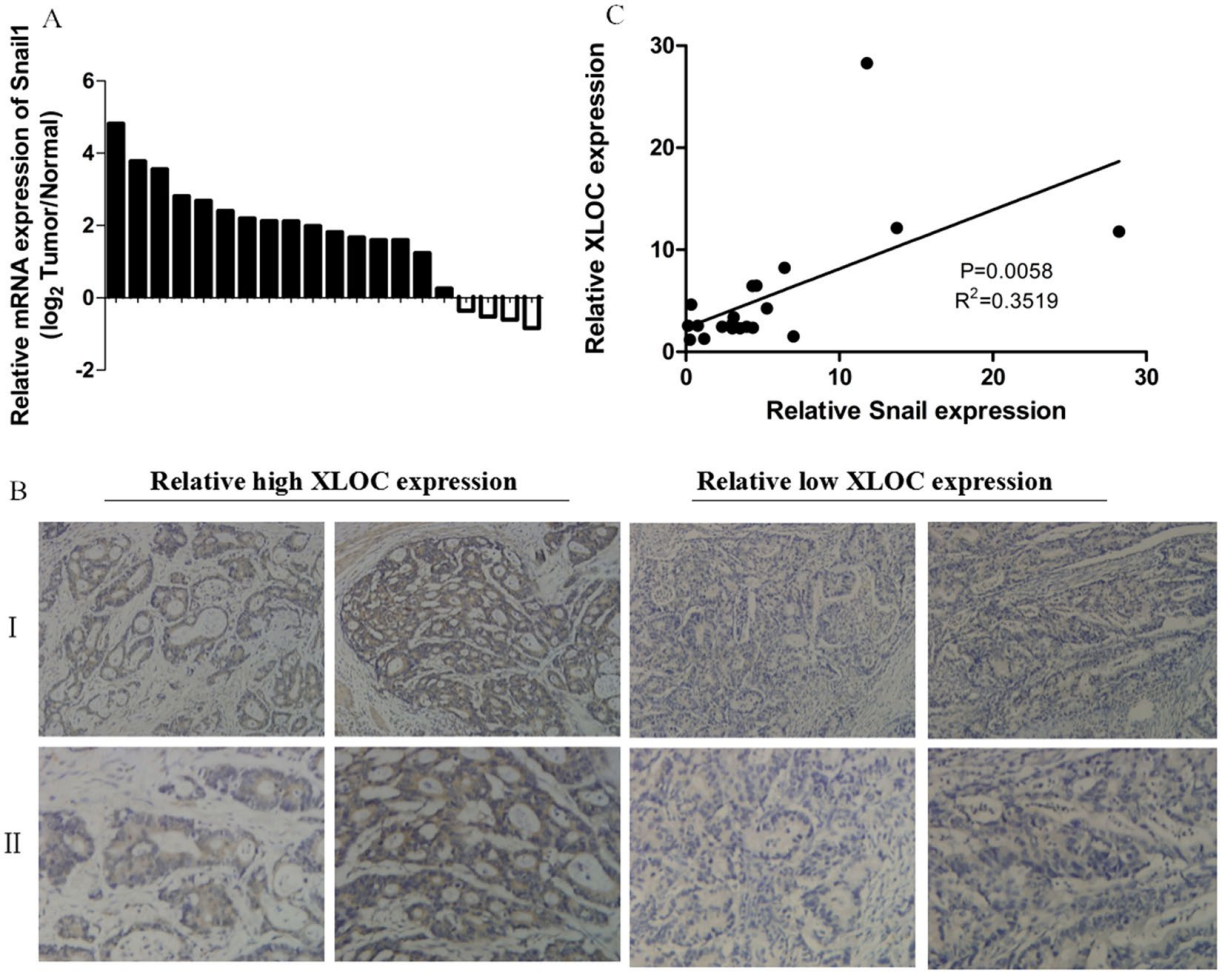
Positive correlation between XLOC_010235 and Snail1 expression. (**A**) Snail1 mRNA levels in gastric cancer tissues were analyzed by qRT-PCR. (**B**) Immunostaining of Snail1 protein in gastric cancer tissue samples. Left panel: immunostaining of Snail1 was positive in gastric cancer tissues with relatively high XLOC expression. Right panel: immunostaining of Snail1 was negative in gastric cancer tissues with relatively low XLOC expression. Original magnifications of ×200 (I) and ×400 (II) are shown. (**C**) Analysis of the relationship between XLOC expression and Snail1 mRNA level in 20 gastric cancer tissues.

To assess the relationship between XLOC and Snail1 expression in gastric cancer, we detected expression of Snail1 in 20 paired gastric cancer tissues by immunohistochemistry (IHC) and qRT-PCR analysis. Compared with the levels in matched normal tissues, the mRNA levels of Snail were generally higher in gastric cancer tissues (Fig. 4A). The results of IHC staining showed 56% of Snail1 protein positive cancer tissues displayed high XLOC expression (Fig. 4B, Supplementary file 4: Table S3). Further analysis showed that Snail1 expression was significantly correlated with XLOC transcript level in gastric cancer tissues (Fig. 4C, Supplementary file 4: Table S3). These data indicated that the expression of Snail1 is positively associated with up-regulated XLOC in gastric cancer tissue samples.

## Discussion

Recent evidence has shown that ncRNAs play an important role in cancer pathophysiology, and could provide new avenue into the biology of this disease^18, 21^. In our previous study, we focus mainly on the clinical significance and indicative function for metastasis development of aberrant expression of XLOC_010235 with specific mechanism unclarified^19^.

As high XLOC expression was associated with a metastatic and aggressive tumor phenotype, we presumed that XLOC could play a significant role in the biological process of metastasis. In current study, we initially demonstrated that ectopic expression of XLOC led to the significant promotion of cell viability, migration and invasion whereas knockdown of XLOC expression impeded these activities. These findings indicate that XLOC exerts an marked influence on the regulation of GC cell progression and metastasis, and may be available as a novel metastatic or prognostic marker for gastric cancer.

Previously, many tumor-associated lncRNAs have been described^22^ and the roles of these RNAs in the development and progression of cancer is being studied^18, 23, 24^. However, literature regarding the involvement of lncRNAs related to metastatic progression in GC is still scarce.

Our observation that XLOC_010235 is up-regulated in GC cell lines was parallel to that of a previous study^25^ in which up-regulation of XLOC_013014 was associated with a poor prognosis in patients with esophageal squamous cell carcinoma. Also, down-regulation of XLOC_010588 is thought to promote proliferation and indicate a poor prognosis in cervical cancer^26^. Moreover, Feldstein Q *et al*. have revealed that XLOC_006942 restricts apoptosis induced by E2F1 and by DNA damage^27^. Taken together, these findings suggest that altered expression of XLOC family members exerts significant effects on the development of various types of tumors and indicates a relatively poor prognosis.

Epithelial to mesenchymal transition (EMT) are characterized by the loss of E-cadherin expression, and ectopic expression of N-cadherin and Vimentin^28–31^. Therefore, the protein levels of these EMT-induced markers were detected following XLOC depletion or over-expression compared to controls, respectively. Our results demonstrated that stimulative effects on cell migration and invasion that were associated with EMT. Matrix metalloproteases (MMPs) are well known to play essential roles in invasion and metastasis in human carcinomas^32, 33^. Up-regulation of XLOC expression in GC cells led to a significant increase in MMP2/MMP9 protein levels. Our findings indicated that XLOC mediated GC cell migration, invasion and metastasis promotion, which possibly also affected EMT.

To explore the molecular mechanism through which XLOC contributes to the GC metastasis and invasion, we explored potential target proteins involved in cell motility and matrix invasion. We identified which molecules were differentially expressed after over-expression or blocking of XLOC, compared with untreated cells, mRNA levels of Snail1 were reduced or elevated upon the aberrant expression or depletion of XLOC, respectively. Western blot analysis was carried out to confirm that Snail1 protein levels were similarly regulated by XLOC, which indirectly confirmed that Snail1 may be positively regulated by XLOC. Snail is known as a family of transcription factors that promote the repression of the adhesion molecule E-cadherin to regulate EMT^34^. Snail1 binds to E-box, an E-cadherin promoter region^34^, and represses the expression of the adhesion molecule, which induces the tightly bound epithelial cells to break loose from each other and migrate into the developing embryo to become mesenchymal cells. The expression of Snail1 in various cancers were usually associated with progression and metastasis^35–37^. Moreover, Snail was implicated in functional mechanism of numerous molecules in modulating EMT and even served as a direct target. For instance, Zuo QF *et al*. found that microRNA-22 inhibited GC cell metastasis by directly targeting Snail^38^. In view of explicit function and mechanism about effect of Snail1 on cancer progression, metastasis and invasion, functional assays including migration and invasion assays of Snail1 alone were not implemented in the study.

We showed that co-transfection of si-Snail1 could availably alleviate the metastatic phenotype and suppress the EMT process. Simutaneously, the co-transfection of pcDNA -XLOC absolutely exerts influence on the promotion of EMT compared to the alone transfection of si-Snail1, indicating that XLOC regulated EMT partly through Snail1. However, further investigation will be necessary to verify the precise molecular mechanisms how XLOC interacted with Snail1 to influence EMT procedure.

## Conclusions

In summary, our study showed that up-regulation of XLOC has the effect of accelerating gastric cancer cell migration and invasion *in vitro*. Moreover, Snail1 was closed involved in the XLOC mediated promotion to EMT. However, adequate disclosure on specific mechanism of XLOC regulating EMT via Snail1 may helpful to the treatment of gastric cancer.

## Methods

### Cell lines

Six gastric adenocarcinoma cell lines (AGS, BGC-823, BGC-803, SGC-7901, HGC-27, MKN-1) and the normal human epithelial cell line (GES-1) were purchased from the Institute of Biochemistry and Cell Biology of the Chinese Academy of Sciences (Shanghai, China). AGS, BGC-823, BGC-803, HGC-27, MKN-1 cells were cultured in RPMI 1640; SGC-7901 and GES-1 cells were cultured in DMEM (GIBCO-BRL) medium supplemented with 10% fetal bovine serum (FBS), 100 U/ml penicillin and 100 mg/ml streptomycin (Invitrogen, Carlsbad, CA, USA), and maintained at 37 °C with 5% CO_2_.

### RNA extraction and Quantitative real-time PCR

Total RNA was isolated with TRizol reagent (Invitrogen) according to the manufacturer’s instructions. the reverse transcription reactions were performed using random primers. Real-time PCR was carried out using a standard SYBR Green PCR kit (TaKaRa, Dalian, China) protocol on Applied Biosystems 7300 Real-Time PCR system (Applied Biosystems). Results were normalized to the expression of glyceraldehyde-3-phosphate dehydrogenase (GAPDH). The specific primers used are presented in Additional file 1. (Table S1) Each sample was assayed in triplicate. The comparative threshold cycle method was used to calculate the relative expression. The relative fold change in gene expression was calculated by using the following formula: ${2}^{-{\rm{\Delta }}{\rm{\Delta }}\text{Ct}}={2}^{-[{\rm{\Delta }}\text{Ct}({\rm{tumor}}{\rm{samples}})-{\rm{\Delta }}\text{Ct}({\rm{vehicle}}{\rm{control}})]}$, where ΔCt = Ct (detected gene)–Ct(β-actin) and Ct represents the threshold cycle number.

### Vector construction and transfection, and siRNA transfection

The XLOC sequence was synthesized and subcloned into the pcDNA3.1 (Invitrogen, Shanghai, China) vector. Ectopic expression of XLOC was achieved through pcDNA-XLOC transfection, with an empty pcDNA3.1 vector used as a control. To generate XLOC knockdown MKN-1 cells, the target sequence for XLOC siRNA or scrambled siRNA that did not correspond to any human sequence was synthesized by Invitrogen. To generate Snail1 knockdown MKN-1 cells, the target sequence for Snail1 siRNA was transfected into the cells, using Lipofectamine 3000 (invitrogen), accordingto the manufacturer’s instructions. The sequence of XLOC, siRNA-XLOC were shown in Additional file 2. (Table S2) The expression levels of XLOC were detected by q-PCR.

### Cell viability assays

Cell viability was monitored using a Cell Counting Kit (CCK) −8 (Dōjindo Laboratories). The MKN-1 cells transfected with si-XLOC (3000 cells/well), and SGC-7901 cells transfected with pCDNA-XLOC were grown in 96-well plates. Cell viability was assessed every 24 h following the manufacturer’s protocol. All experiments were performed in quintuplicate.

### Wound-healing assay

For the wound-healing assay, 3 × 10^5^ cells were seeded in 6-well plates, cultured overnight, and transfected with pCDNA-XLOC, si-XLOC or a control. Once cultures reached 85% confluence, the cell layer was scratched with a sterile plastic tip and washed with culture medium, then cultured for 48 h with medium containing 1% FBS. At different time points, images of the plates were acquired using a microscope. The distance between the two edges of the scratch was measured using Digimizer software system. The assay was independently repeated three times.

### Cell migration and invasion assays

For the migration assays, at 48 h post-transfection, 5 × 10^4^ cells in serum-free media were placed into the upper chamber of an insert (8-μm pore size; Millipore). For the invasion assays, 1 × 10^5^ cells in serum-free medium were placed into the upper chamber of an insert coated with Matrigel (BD Bioscience). Medium containing 10% FBS was added to the lower chamber. After incubation for 24 h, the cells remaining on the upper membrane were removed with cotton wool. Cells that had migrated or invaded through the membrane were stained with methanol and 0.1% crystal violet, imaged, and counted using an IX71 inverted microscope (Olympus, Tokyo, Japan). Experiments were independently repeated three times.

### Western blotting analysis

Cells were lysed using RIPA protein extraction reagent (Beyotime, Beijing, China) supplemented with a protease inhibitor cocktail (Roche, CA, USA) and phenylmethylsulfonyl fluoride (Roche). The concentration of proteins was determined using the Bio-Rad protein assay kit. Protein extracts (50 μg) were separated by 10% sodium dodecyl sulfate-polyacrylamide gel electrophoresis (SDS-PAGE), then transferred to nitrocellulose membranes (Sigma) and incubated with specific antibodies. ECL chromogenic substrate was used to visualize the bands and the intensity of the bands was quantified by densitometry (Quantity One software; Bio-Rad), with GAPDH used as a control. Antibodies (1:1000 dilution) against E-cadherin and N-cadherin were purchased from BD. Antibodies against Vimentin, MMP-2, and MMP-9 were purchased from Cell Signaling Technology (MA, USA).

### Fluorescence immunohistochemistry

Cells were fixed in 4% paraformaldehyde following a standard protocol. Mouse anti-E-cadherin and -N-cadhherin polyclonal antibodies (1:100; BD) were used as primary antibodies, with TRITC-labeled anti-Rabbit IgG (1:200; Sigma) used as a secondary antibody. Sections were mounted onto slides using Gel Mount Aqueous Mounting Medium (G0918, Sigma) and examined with an OlympusBX51 microscope (Olympus Optical, Tokyo, Japan).

### Statistical analysis

Student’s t-test (2-tailed), one-way ANOVA, and the Mann–Whitney U test were used to analyze data, along with SPSS 16.0 (IBM, IL, USA). P-values of less than 0.05 were considered statistically significant.

## Electronic supplementary material

Supplementary information
