# Supplementary material for: Up-regulation of long non-coding RNA XLOC_010235 regulates epithelial-to-mesenchymal transition to promote metastasis by associating with Snail1 in gastric cancer
Source: Sci Rep. 2017 May 26;7:2461. doi: 10.1038/s41598-017-02254-6 (PMC5446413; doi:10.1038/s41598-017-02254-6)
Supplement: Supplementary file 1 — Supplementary information [file 41598_2017_2254_MOESM1_ESM.pdf]

Up-regulation of long non-coding RNA XLOC_010235 regulates epithelial-to-mesenchymal transition to promote metastasis by associating with Snail1 in gastric cancer

Yu-yi Liu[1+], Ze-hong Chen[1+], Jian-jun Peng[1], Jia-lin Wu[1], Yu-jie Yuan[1], Er-tao Zhai[1], Shi-rong Cai[1], Yu-long He [1*], Wu Song[1*]

+Equal contributor * Corresponding author

1 Department of Gastrointestinal Surgery, The First Affiliated Hospital, Sun Yat-sen University, Zhongshan Second Road 58, Guangzhou, 510080, Guangdong Province, China

*Corresponding author:

Wu Song, Department of Gastrointestinal Surgery, The First Affiliated Hospital, Sun Yat-sen University, Zhongshan Second Road 58, Guangzhou, 510080,China songwu@mail.sysu.edu.cn

Telephone: +86-20-28823350 Fax: +86-20-28823389

Yu-long He, Department of Gastrointestinal Surgery, The First Affiliated Hospital, Sun Yat-sen University, Zhongshan Second Road 58, Guangzhou, 510080,China ylh@medmail.com.cn.

Telephone: +86-20-28823350 Fax: +86-20-28823389

Supplementary File 1: Table S1. Primers used for qRT-PCR and siRNAs oligonucleotides

	sequence(5'-3')
XLOC_010235-F:	ATGCTGGAGTTTGGATCATATTCC
XLOC_010235-R	CGTCCTTCTTTGTCTCACTTCAC
Snail1-F	GCACATCCGAAGCCACAC
Snail1-R	GGAGAAGGTCCGAGCACA
CD44-F	CTGCCGCTTTGCAGGTGTA
CD44-R	CATTGTGGGCAAGGTGCTATT
Integrinbeta 1-F	CCTACTTCTGCACGATGTGATG
Integrinbeta 1-F	CCTTTGCTACGGTTGGTTACATT
ICAM-1-F	GTGACCAGCCCAAGTTGTTG
ICAM-1-R	AGTCCAGTACACGGTGAGGA
FOXF1-F	CACTCCCTGGAGCAGCCGTATC
FOXF1-R	AAGGCTTGATGTCTTGGTAGGTGA
E-Cadherin-F	TGCCTGAGAACGAGGCTAAC
E-Cadherin-R	TGGGGGCTTCATTCACATCC
N-cadherin-F	TCTGGGTCTGTTTTATTACTCCTGG
N-cadherin-R	CGAGCTGATGACAAATAGCGG
Vimentin-F	AACTTAGGGGCGCTCTTGTC
Vimentin-R	CCTGCTGTCCCGCCG
MMP-2 F	CGTCTGTCCCAGGATGACATC
MMP-2 R	TGTCAGGAGAGGCCCCATAG
MMP-9 F	TGGGCAGATTCCAAACCTTT
MMP-9 R	TCTTCCGAGTAGTTTTGGATCCA
GAPDH-F	GCTCTCTGCTCCTCCTGTTC
GAPDH-R	ACGACCAAATCCGTTGACTC
scrambled	UUCUCCGAACGUGUCACGUTT

Supplementary File 2: Table S2. The sequence of lncRNA XLOC_010235 and siRNA-XLOC_010235

	sequence(5'-3')
XLOC_010235	GAACTCTCTGGGCCCCTGCGATGTAGGTTGCCTAAGGCAGCTGTCTTGGGAGTGATGCTG GAGTTTGGATCATATTCCATGCGCCCGTGGCTCCAAATGCCCATCATTCCTCAGTACTGG GCAAAGGTTGTTCAGCCTCAGGTGAAGTGAGACAAAGAAGGACGTCAAGAGGTTTTGCTG GGTTTGATGGGTTTCATCTGGGACGGGTTACAATGCATTTTGTGTAATCATCTCTTTTCA TTAAGTGGCAGGCATTGTAGATACAGTTCAGTCATTCTTTTTTATGGCATGTTTAGTTGG CA
si-XLOC_010235-1	GGUUUGAUGGGUUUCAUCU dTdT

si-XLOC_010235-2 UCAUUAAGUGGCAGGCAUU dTdT

si-XLOC_010235-3 GCAUUGUAGAUACAGUUCA dTdT

Supplementary File 3: Figure S1. Knockdown efficiency of siRNA-Snail1

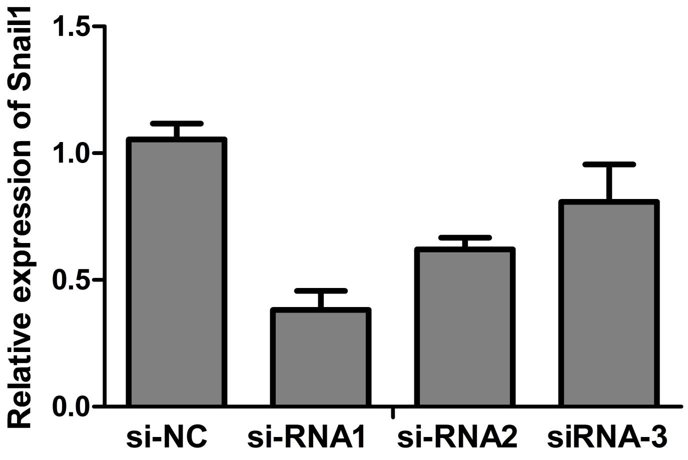

Supplementary File 4: Table S3. XLOC relative expression level and Snail1 sores of immunohistochemistry

Patient No.	XLOC expression（ΔΔCt value)	Snail1scores(IHC)
1	1.2798	2
2	2.3554	4
3	2.55265	0
4	2.4539	4
5	2.47255	2
6	4.6267	0
7	6.4513	4
8	1.1974	0
9	2.566	0
10	3.3755	3
11	2.3195	3
12	11.7941	9
13	2.7411	3
14	6.498	6
15	8.23	6
16	2.3133	2
17	4.2657	4
18	12.1257	9

19	28.296	9
20	1.51571	1
